# Aging Behavior and Durability of Polymer Materials, 2nd Edition

**DOI:** 10.3390/polym18192320

**Published:** 2026-09-23

**Authors:** Ivana Salopek Čubrić, Goran Čubrić

**Affiliations:** Department of Textile Design and Management, University of Zagreb, 10000 Zagreb, Croatia; goran.cubric@ttf.unizg.hr

Polymeric materials are indispensable in modern society, finding applications in textiles, construction, transportation, electrical engineering, infrastructure, and many other fields. Their long-term performance, however, can be affected by environmental exposure, mechanical loading, temperature, moisture, radiation, and chemical agents, making an understanding of aging and durability essential for their safe and reliable use. Polymer aging is a complex process influenced by environmental conditions such as temperature, humidity, chemical exposure, and mechanical or thermal stresses, which can progressively alter material structure and performance [1,2]. Studies on polypropylene have shown that aging in detergent solutions can modify surface properties and consequently affect the interaction of polymer surfaces with microorganisms, demonstrating that aging may influence not only mechanical performance but also surface functionality [1]. Similarly, hydrothermal exposure can cause significant degradation of epoxy-based materials, particularly those used for electrical insulation, highlighting the importance of understanding the relationship between environmental conditions, material composition, and long-term functional stability [3]. Reliable prediction of polymer service life therefore requires appropriate accelerated-aging methodologies and predictive models, such as time–temperature equivalence approaches, which can be used to estimate natural aging behavior from accelerated testing [4].

The Special Issue “Aging Behavior and Durability of Polymer Materials, 2nd Edition” brings together contributions addressing different aspects of polymer aging, degradation, functional stability, durability assessment, and long-term performance prediction. The studies cover a broad range of materials and applications and demonstrate the importance of combining structural characterization, accelerated aging, performance testing, and predictive approaches.

The first published contribution investigates the long-term aging of historical lignocellulosic materials. Bouramdane and coworkers examined cedar wood from the 9th and 12th centuries using FTIR-deconvolution spectroscopy, X-ray diffraction, crystallinity analysis, and SEM [5]. The results revealed changes in cellulose crystallinity, lignin structure, and morphology associated with long-term biological degradation. The study provides a unique perspective on polymer aging over centuries and demonstrates the value of complementary analytical techniques for understanding structural deterioration. Another contribution focuses on the environmental exposure of conventional and recycled polyester sportswear [6]. Salopek Čubrić et al. evaluated changes in thickness, porosity, elongation, bending stiffness, abrasion resistance, and wetting behavior. Environmental exposure affected several properties, while recycled polyester showed particularly favorable abrasion resistance after aging. These findings emphasize the importance of assessing durability through a range of properties relevant to actual applications. Li et al. investigated hydrothermal aging and long-term creep behavior of polyvinyl chloride [7]. Accelerated aging at 60 °C and 90% relative humidity was combined with mechanical, spectroscopic, microscopic, and structural analyses. The authors applied time–stress and time–aging equivalence principles to predict long-term performance. The study highlights the potential of combining accelerated aging with modeling to estimate polymer service life over experimentally inaccessible timescales. The following contribution addresses the aging of rubber sealing strips used in hydropower equipment [8]. Chen et al. proposed an aging-feature-extraction method based on the ratio between the short-axis and original dimensions of serviced rubber strips. The relationship between this geometric parameter and mechanical properties provides a practical approach for evaluating the aging state of rubber components after long-term service. Hu et al. investigated the influence of wax-based warm-mix additives on the rheological and aging behavior of high-viscosity polymer-modified asphalt [9]. By combining rheological, spectroscopic, and microscopic analyses, the authors demonstrated that additive selection can significantly affect both processing and aging resistance. Refined Fischer–Tropsch wax showed particularly promising performance, illustrating how durability can be improved through material formulation. Tarbuk et al. investigated cotton/polyester fabrics modified through enzymatic treatment and chitosan functionalization [10]. The study examined surface, interfacial, mechanical, spectral, and antimicrobial properties, with particular attention to the stability of the functional treatment during washing. The retention of chitosan after repeated washing demonstrates the potential of enzymatic activation for producing more durable functional textile surfaces. The contribution by Ming et al. examines damp-heat aging of epoxy and phenolic resin insulation materials [11]. Interestingly, aging at 85 °C and 90% relative humidity improved the measured flame-retardant properties of both systems. The observed changes were associated with alterations in surface composition and the formation of protective barrier layers. These findings demonstrate that aging does not necessarily result in deterioration of every material property and that its effects must be evaluated according to the specific degradation mechanisms involved. The most recently published contribution, by Bae et al., investigates the long-term aging of closed-cell phenolic foam insulation [12]. The authors identified a transient minimum in thermal conductivity, with an initial decrease followed by an increase during aging. This phenomenon was attributed to the redistribution of n-pentane between the polymer matrix and cell-gas phase, followed by its gradual replacement by air. The study demonstrates the complex interaction between polymer structure, cell morphology, gas diffusion, and long-term thermal performance.

Taken together, these contributions demonstrate that polymer aging is a complex, material-specific, and multiscale phenomenon. The studies encompass centuries-long natural degradation, accelerated aging and lifetime prediction, in-service condition assessment, formulation-based improvement of aging resistance, and the evaluation of functional stability. They also show that aging may affect different properties in different ways and, in some cases, may even improve selected characteristics. Overall, the Special Issue highlights the importance of linking molecular and morphological changes with macroscopic performance and practical service requirements. Future research should increasingly address coupled aging mechanisms, realistic exposure conditions, in situ monitoring, and predictive approaches capable of translating accelerated laboratory testing into reliable service-life estimates. Such developments will support the design of safer, more durable, and more sustainable polymeric materials.
